# Ten tips for how to prevent and treat hemodynamic instability during hemodialysis

**DOI:** 10.1093/ckj/sfag358

**Published:** 2026-10-08

**Authors:** Sebastian Mussnig, Panagiotis I Georgianos, Manfred Hecking, Daniel Schneditz, Yuri Battaglia, Christian Combe, Vassilios Liakopoulos, Casper Franssen

**Affiliations:** Medical University of Vienna, Department of Medicine III, Division for Nephrology & Dialysis, Vienna, Austria; 2nd Department of Nephrology, AHEPA Hospital, School of Medicine, Aristotle University of Thessaloniki, Thessaloniki, Greece; Medical University of Vienna, Department of Medicine III, Division for Nephrology & Dialysis, Vienna, Austria; Medical University of Graz, Otto Loewi Research Center for Vascular Biology, Immunology and Inflammation, Division of Physiology & Pathophysiology, Graz, Austria; Center for Medical Science, University of Trento, Trento, Italy; Multidistrict Nephrology and Dialysis Unit, ASUIT, Trento, Italy; Bordeaux School of Public Health, Université de Bordeaux, INSERM U1219, CIC 1401, Bordeaux, France; Department of Nephrology and Nutrition, Hospices Civils de Lyon, Centre Hospitalier Lyon-Sud, Pierre-Bénite, France; Centre Hospitalier d’Ardèche Méridionale, Aubenas, France; 2nd Department of Nephrology, AHEPA Hospital, School of Medicine, Aristotle University of Thessaloniki, Thessaloniki, Greece; Department of Nephrology, University Medical Center Groningen, University of Groningen, Groningen, The Netherlands

**Keywords:** blood pressure, chronic hemodialysis, dry weight, hypotension, intradialytic hypotension

## Abstract

Intradialytic hypotension (IDH) is a frequent complication of hemodialysis that may occur with symptoms and often requires interventions to restore hemodynamic stability and end-organ perfusion. In the absence of large trials with IDH as the primary endpoint, guidelines recommending specific management protocols are not available, but hemodynamic instability is a daily clinical reality, nevertheless. First, we suggest defining and monitoring IDH, by adopting a uniform IDH definition such as the Nadir90 criteria and document symptoms, and regularly measuring intradialytic as well as ambulatory blood pressure. Next, we give recommendations on how to treat IDH, by developing a standard operating procedure for acute events, and increasing dialysate sodium, and decreasing ultrafiltration rates and dialysate temperature. Finally, the majority of our tips are dedicated to preventing IDH, by regularly assessing fluid status, reviewing antihypertensive drugs, considering more frequent, longer or nocturnal dialysis, potentially considering hemodiafiltration, avoiding food and considering exercise, and trying automatic feedback algorithms. No single measure consistently prevents IDH, and each tip should be viewed as one lever among many. In practice, IDH is best managed by a layered, individualized approach that combines rigorous documentation, structured follow-up and a willingness to revisit the prescription each time hemodynamic instability recurs.

## INTRODUCTION

Intradialytic hypotension (IDH) is a frequent complication of dialysis that may occur with symptoms and often requires interventions to restore hemodynamic stability and end-organ perfusion [1, 2]. For some time, hemodynamic instability was considered a sign that “dry weight” had been achieved [3]. However, several large observational studies have identified IDH as a strong independent risk factor for vascular access thrombosis, cardiovascular events and mortality [4–7]. A more balanced approach to fluid management is now recommended [8, 9]. Given the growing attention to patient symptoms [10–12] and the continued goal to reduce excess cardiovascular mortality in hemodialysis [13], systematic assessment and treatment of intradialytic hemodynamic instability are crucial. However, chronic fluid overload (FO) must be avoided [14], and management of IDH may consequently require individualized approaches.

In the absence of large trials with IDH as the primary endpoint, it is difficult to formulate universal management recommendations. This does not change the reality that clinicians encounter intradialytic hemodynamic instability on a daily basis. The aim of this article is neither to review mechanisms causing hemodynamic instability—this has been addressed comprehensively elsewhere (Table 1)—nor to provide a systematic account of established techniques. Instead, we present a series of practical tips grounded in clinical experience and available literature, to aid the management of hemodynamic instability among hemodialysis patients. Tips are ordered by decreasing urgency and are grouped into measures to define and monitor IDH, treat IDH and prevent IDH (Fig. 1). Because the evidence supporting these tips ranges from randomized controlled trials to physiological reasoning and clinical experience, each tip closes with a short statement of its evidence base. We have deliberately not applied a formal grading system, which would suggest a systematic appraisal that this article does not provide.

**Figure 1:**
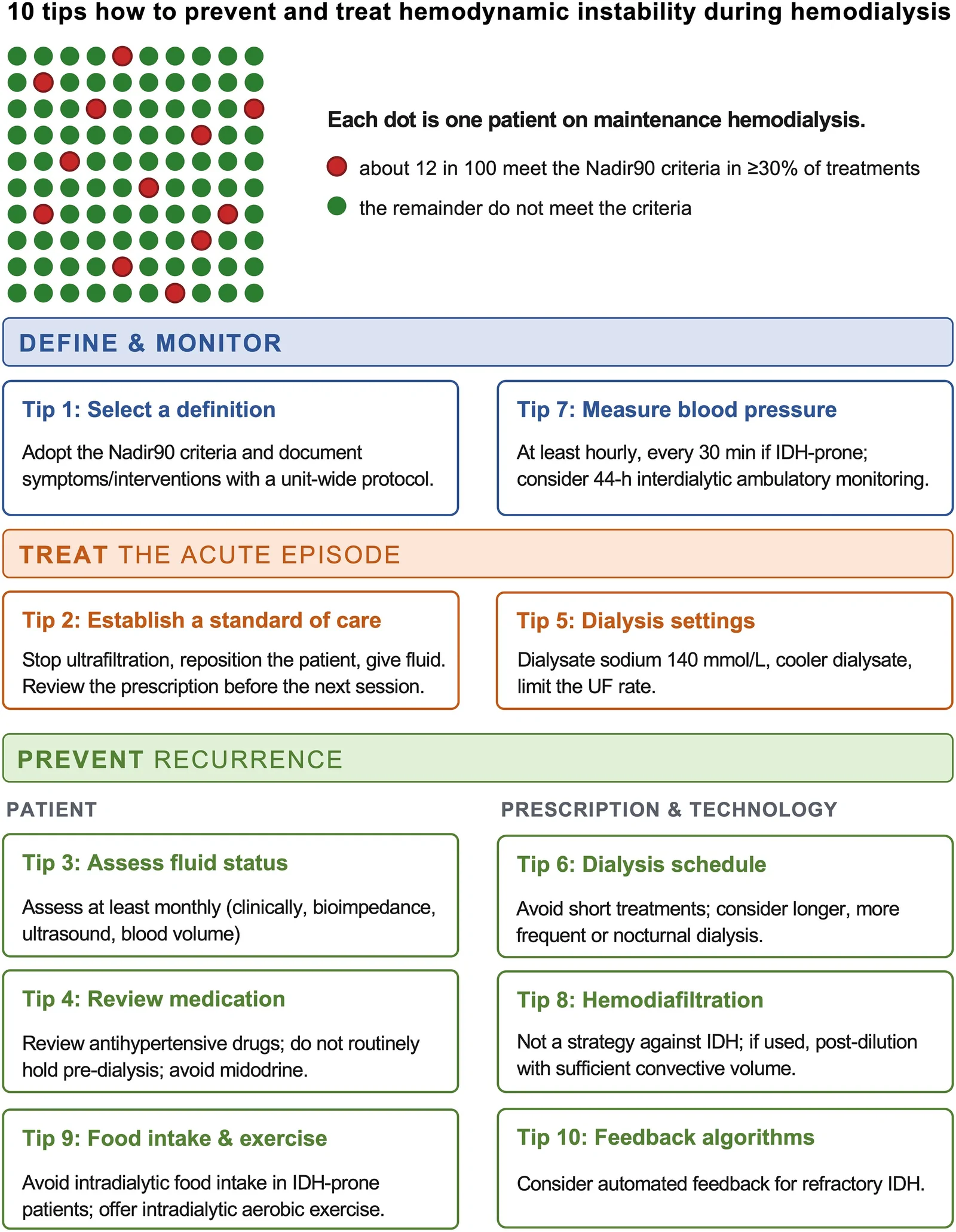
Overview over the proposed tips. Tips to define and monitor, treat and prevent intradialytic hypotension. IDH, intradialytic hypotension; UF, ultrafiltration.

**Table 1:** Summary of recent review articles on the topic of IDH.

Author (year)	Focus	Key messages	Differences from the present 10 tips article
Reeves and McCausland (2018) [130]	Mechanisms, outcomes, treatment	Whole plasma volume removal thrice weekly exceeds normal hemodynamic compensatory reserve in many patients IDH is independently associated with higher cardiovascular and all-cause mortality Prevention combines UFR limitation, dialysate cooling, sodium management and targeted pharmacotherapy (e.g. midodrine)	Comprehensive on the individual therapeutic options but does not prioritize or sequence them for the individual patient, nor separate acute from long-term measures
Sars *et al.* (2020) [131]	Mechanisms and outcome	IDH reflects the interaction of blood-volume decline, impaired vasoreactivity and limited cardiovascular reserve Absolute blood-volume measurement (online dilution) outperforms relative blood-volume monitoring for predicting IDH Isothermic/cool dialysis reduces end-organ damage, though a mortality benefit has not yet been demonstrated	Detailed on volume physiology and monitoring technology but offers no framework for managing the acute episode or the interdialytic period
McIntyre and Goldsmith (2015) [132]	IDH and ischemic brain injury	Repeated IDH drives cerebral hypoperfusion, white-matter injury and brain atrophy These ischemic insults underpin the high prevalence of cognitive decline and depression in hemodialysis patients Dialysis-technique modifications (cooler dialysate, lower UFR) may mitigate brain injury—a landmark shift from a purely uremic paradigm	Establishes IDH as an end-organ injury rather than a benign event, but does not address how IDH should be recognized, recorded or acted upon in routine care
Chou *et al.* (2017) [133]	Epidemiology and survival	Nadir SBP <90 mmHg during >30% of sessions is the definition most consistently linked to mortality UFR >10 mL/h/kg (and especially >13 mL/h/kg) predicts cardiovascular and all-cause death IDH mediates repeated myocardial stunning, stroke risk and gut endotoxin translocation—calling for a paradigm shift	Identifies which definition best predicts outcomes but stops short of translating this into a documentation and monitoring standard that a dialysis unit can adopt
Davenport (2023) [134]	Modern prescription and technology	Nadir SBP is the IDH metric most tightly linked to outcomes Risk is shaped by patient comorbidity, postdialysis target weight, dialysate composition and UFR profile Real-time volume- and tonicity-sensing biofeedback is promising but not yet sophisticated enough to reliably prevent IDH	Reviews the components of the prescription individually; does not address unit-wide standardization or the order in which measures should be tried in a patient who keeps dropping
Kanbay *et al.* (2020) [135]	Definitions and research agenda	IDH results from excess UFR against inadequate cardiac output and arteriolar tone End-organ hypoperfusion affects heart, brain, kidney and gut—arguing for an outcome-based definition Proposes a research roadmap covering dialysis prescription, intra-session interventions and interdialytic management	Directed primarily at the research community; sets out what should be studied next rather than what the bedside team should do with the evidence available today

SBP, systolic BP; UFR, ultrafiltration rate.

## TIP 1 (DEFINE AND MONITOR): SELECT A DEFINITION FOR INTRADIALYTIC HYPOTENSION THAT FITS YOUR CENTER’S PRACTICE, AND MONITOR ROUTINELY

Definitions of IDH are based on either a decline in blood pressure (BP) from pre-dialysis (e.g. by 20 mmHg), a minimum BP threshold or nadir (e.g. below 90 mmHg), clinical interventions (e.g. fluid infusion, Trendelenburg position, changes to blood flow or ultrafiltration), patient-reported symptoms (e.g. dizziness, nausea, cramps) or a composite of multiple criteria [15]. In the 2000s, European [16], American [17] and Japanese [18] nephrological societies adopted definitions based on BP drops with symptoms or interventions, without clear supporting evidence.

In an analysis of two large hemodialysis populations (HEMO) (*n* = 1409) and a large dialysis organization (LDO) (*n* = 10 392), Flythe *et al.* [7] compared different IDH definitions and their associations with mortality. The Nadir90 criteria, where IDH is defined by a drop in systolic BP below 90 mmHg in 30% of treatments, was met in 9.7%–11.3% of patients and exhibited the only statistically significant association with mortality in adjusted analyses [odds ratio (OR) in HEMO for 2-year mortality: 1.56, 95% confidence interval (CI) 1.05–2.31; OR in LDO for 1-year mortality: 1.30, 95% CI 1.07–1.57; and also in another observational study: hazard ratio (HR) for Nadir90 in 40% vs 0% of treatments 1.49, 95% CI 1.42–1.57 [19]]. This association increased with the frequency of IDH-afflicted treatments. Adding interventions or symptoms eliminated statistical significance, which the authors attributed to large variability in symptom reporting and the lack of a standardized intervention protocol [7]. In a secondary stratified analysis of patients with pre-dialysis BP ≥160 mmHg, intradialytic BP drops below 100 mmHg in 30% of treatments (Nadir100) showed a strong association with 1-year mortality as well [OR 1.29 (1.07–1.56)] [7]. A KDIGO controversies conference on “BP and volume management in dialysis” [9] later embraced the Nadir90 criteria, but added that “any symptomatic decrease in systolic BP” warrants reassessment.

Hence, we suggest (i) adopting the Nadir90 criteria if the routine BP measurement frequency and data infrastructure allow for it and (ii) evaluating the frequency of treatments complicated by IDH in regular intervals to identify hypotension-prone patients. In the absence of validated instruments for IDH-related symptoms, we recommend (iii) implementing a standard operating procedure in-center for the documentation of clinical events and interventions associated with IDH, which may help to identify IDH-prone patients.

### Evidence level

The evidence base for this tip is large observational analyses comparing candidate definitions against mortality, supported by a KDIGO consensus statement. No trial has compared the use of one definition against another, and the choice of a threshold remains, ultimately, a pragmatic one.

## TIP 2 (TREAT): ESTABLISH A STANDARD OF CARE FOR ACUTE HEMODYNAMIC INSTABILITY

A standardized approach is also essential for effective management of acute intradialytic hemodynamic instability, which is nearly always symptomatic (e.g. muscle cramps, nausea, vomiting, abdominal pain, acute dyspnea, thoracic pain, neurological symptoms including loss of consciousness and occlusion of the arteriovenous vascular access) [9, 11].

Treatment of acute IDH aims to rapidly increase BP and ensure perfusion of vital organs. Since hypovolemia plays an important role in the pathophysiology of IDH, most of the measures are aimed at increasing the blood volume. The (temporary) cessation of ultrafiltration and intravenous administration of physiological saline (e.g. a bolus of 200 mL) or albumin (bolus of 100 mL) induce a rapid but modest increase in relative blood volume [16]. Infusions of hypertonic mannitol, 50% glucose and hypertonic saline are all able to promptly increase BP [20]. Placing the patient in the Trendelenburg position increases the venous return to the heart. As a result, one can expect the filling pressure in the left ventricle and the cardiac output to rise. However, detailed data on the effectiveness of the Trendelenburg position are scarce [22]. There is no proof that reducing blood flow and/or dialysate flow is an effective measure to restore hemodynamic stability [21]. In most patients, the above-mentioned measures rapidly increase the BP and reverse the symptoms. However, possible non-fluid-related causes of hypotension must be investigated as well, especially in case of refractory and/or recurrent hypotension. These other causes may include myocardial infarction, untreated heart failure, cardiac arrhythmias [23, 24], hemorrhage (e.g. in the digestive tract), bacteremia/sepsis and dialyzer reactions.

Once a patient has experienced an episode of IDH, it is important to prevent future recurrences. All aspects of the individual dialysis prescription should be reviewed periodically and adapted where necessary. Importantly, the patient’s perspective should also be considered, e.g. by repeated patient-reported outcome measures and by asking the patient how long it takes to recover after a hemodialysis session. This recovery time may capture the overall intra- and interdialytic hemodynamic stress.

### Evidence level

This tip is based predominantly on physiological reasoning and expert consensus. Individual components (osmotic agents, saline, albumin) rest on small physiological studies. For the Trendelenburg position and for reductions in blood or dialysate flow, the evidence is scarce or negative. The value of standardization itself has not been tested.

## TIP 3 (PREVENT): REPEATEDLY ASSESS FLUID STATUS AND ADAPT TARGET WEIGHT

FO [14, 25–27]—excess extracellular fluid—is a direct consequence of most forms of kidney failure. Excessive fluid removal may excessively decrease BP as well. The dependence of BP on blood volume through its effect on cardiac output [28] creates a direct link between target weight and IDH: if the post-dialysis weight is set too low, BP drops are inevitable. Thomson’s and Henderson’s definitions of “dry” weight from 1967 and 1980 describe a “reduction of BP to hypotensive levels during ultrafiltration,” and the “weight obtained at the conclusion of a regular dialysis treatment below which the patient more often than not will become symptomatic and go into shock” [3].

More recent evidence comes from the Dry-Weight Reduction in Hypertensive Hemodialysis Patients (DRIP) trial [29]: hypertensive hemodialysis patients had their antihypertensive medication withdrawn and underwent a stepwise target weight reduction, up to the point where IDH would occur (“probing” of dry weight). In consequence, BP was reduced, but hypotensive episodes, cramps, dizziness and the need to intervene increased markedly (supplement in Agarwal *et al.* [29]). Target weight can today also be determined with several objective measures, like bioimpedance-based volume assessment, besides or on top of clinical probing [30]. Machek *et al.* reported that guiding patients of an entire hemodialysis center towards bioimpedance-based euvolemia “reduced” intradialytic adverse events (including IDH), and improved BP [31]. We have had similar experiences via longitudinal fluid volume assessment by combining BP, bioimpedance and blood volume [32, 33], where most clinical cases (especially those with frequent IDH) appeared logical based on the recorded data.

We acknowledge that there is no randomized evidence assessing IDH as an outcome when practicing a particular target weight approach (versus none). An analysis from the Dialysis Outcomes and Practice Patterns Study (DOPPS) showed that “having a protocol that specifies how often to assess dry weight in most patients in the facility was associated with lower risk of all-cause and cardiovascular mortality” [34]. With the commercialization of technologies to quantify fluid volumes, FO ceased being the elephant in the room that no one could see [35]. Hence, we suggest that overall assessment of “chronic” fluid status should take place as often as possible, but at least every month, to avoid setting target weight either too high (risking chronic FO) or too low (risking IDH).

### Evidence level

This tip is based on observational data (including a facility-level DOPPS analysis), experience and a randomized trial in which stepwise target-weight reduction increased hypotensive episodes. No randomized trial has tested a target-weight assessment strategy with IDH as an endpoint, but observational data suggested that tool-guided fluid management improved IDH.

## TIP 4 (PREVENT): BE CAREFUL WITH ANTIHYPERTENSIVE AND VASOACTIVE MEDICATION

Antihypertensive medications are frequently prescribed for managing hypertension in dialysis [36]. An important issue is whether specific antihypertensive drug categories are superior to others in mitigating the risk of IDH [37]. An observational study emulating a randomized trial analyzed 4072 incident patients on dialysis who initiated antihypertensive medications as a monotherapy [38]. The likelihood of IDH compared with using calcium channel blockers (CCBs) was elevated for β-blockers and α-β blockers (OR 2.27, 95% CI 1.50–3.43), agents blocking the renin–angiotensin system (OR 1.71, 95% CI 1.14–2.57) and diuretics (OR 1.52, 95% CI 1.07–2.16) [38]. These data are important but should be interpreted with caution owing to the study’s observational nature. Accordingly, this study cannot provide direct and causal risk associations to demonstrate the superiority of CCBs over other antihypertensive drug categories in lowering the risk of IDH. Beyond the effects on IDH, the choice of the appropriate antihypertensive therapy in dialysis patients should take into consideration additional and clinically meaningful aspects, such as the BP-lowering benefit, rate control in atrial fibrillation and the cardio-protective efficacy of each antihypertensive drug category.

Patients who are frequently experiencing symptomatic IDH are often advised to hold their antihypertensive medications before dialysis as a measure to assure hemodynamic stability during the dialysis treatment. In a cluster randomized controlled trial (RCT), 10 dialysis units were randomly assigned in a 1:1 ratio either to take (*n* = 66 patients) or to hold (*n* = 66 patients) the BP-lowering medications before dialysis over a follow-up period of 4 weeks [39]. Taking BP-lowering medication before dialysis was not associated with more frequent treatments fulfilling the Nadir90 criteria for IDH in ≥30% of treatments (mean adjusted difference: 8%, 95% CI −3% to 19%) [39]. “Taking” was found to be superior to “holding” for uncontrolled hypertension, defined as a pre-dialysis systolic BP >160 mmHg (adjusted mean difference: −15%, 95% CI −28% to −1%) [39]. In our practice, with the exception of potent and short-acting BP-lowering medications, such as centrally acting antihypertensives or hydralazine, we do not advise our patients to hold their antihypertensive therapy before dialysis as a measure to prevent IDH.

The α-1 adrenergic receptor agonist midodrine is commonly used off-label in clinical practice as a therapeutic approach to mitigate the risk of IDH. However, the safety and efficacy of midodrine has not been demonstrated in properly designed trials [40]. In an observational study, and relative to non-use, the use of midodrine was associated with higher risk of all-cause death, all-cause hospitalization, cardiovascular-related hospitalization, greater likelihood for lower pre-dialysis systolic BP, lower nadir intradialytic systolic BP and higher proportion of dialysis sessions complicated by symptomatic IDH [41], thus its use is not recommended.

### Evidence level

This tip is based on one observational target-trial emulation for drug-class differences, and one small, short, open-label cluster-randomized trial on taking versus holding medication that was not powered for equivalence. Only observational data exist for midodrine, with a safety signal.

## TIP 5 (TREAT): OPTIMIZE DIALYSIS SETTINGS—DIALYSATE SODIUM, ULTRAFILTRATION RATES AND TEMPERATURE?

### Dialysate sodium

A study on 17 patients from 1973 found that individualized dialysate sodium (averaging 149 mEq/L) compared with standard dialysate sodium bath of that time (133 mEq/L) increased systolic BP by 11 mmHg compared with 2 mmHg in controls [42]. Patients in the control and experimental groups experienced an average intradialytic fall in serum osmolality of 26 versus 5.8 mOsm/kg, respectively, accompanied by deteriorating electroencephalogram (EEG) signals (control group) versus very few EEG changes (experimental group), and worse versus better dialysis disequilibrium scores, respectively [43]. This study paved the way for higher dialysate sodium concentrations (as described by Flanigan [43]) and shorter dialysis sessions worldwide. Perhaps in consequence of the fear that sodium “loading” during dialysis would do harm [44], dialysate sodium concentrations were systematically lowered (shown for the international Dialysis Outcomes and Practice Patterns Study, years 1996–2018) [45]. A recent Cochrane meta-analysis of RCTs however found that when compared with neutral or high dialysate sodium (≥138 mEq/L), low dialysate sodium (<138 mEq/L) “probably increases IDH events [13 studies, 15 764 HD sessions: RR 1.58, 95% CI 1.25 to 2.01]” [46] (HD = hemodialysis; RR = relative risk). Hussein and Schiller have discussed potential consequences of interdialytic weight gain with higher dialysate sodium, presenting a pragmatic approach for various scenarios (one of them being: “in patients with frequent IDH, particularly if low interdialytic weight gain, avoid low dialysate sodium, consider dialysate sodium 140 mEq/L or higher”) [47]. A slightly positive dialysate-to-serum sodium gradient can also be administered with recently developed sodium balancing tools (see Tip 10). In any case, changes in dialysate sodium for the sake of treating and preventing IDH should be accompanied by monitoring of intradialytic BP and IDH frequency to evaluate the potential beneficial effects and monitoring of interdialytic weight gain and serum sodium levels to avoid significant diffusive sodium loading. More observational data gathered since Hussein and Schiller [47] have—if anything—challenged the fear of higher interdialytic weight gain (as lower interdialytic weight gain is associated with higher mortality [48]) and of higher dialysate sodium concentrations *per se* (as dialysate dialysate sodium ≤138 mEq/L was associated with higher adjusted mortality than dialysate sodium >138 mEq/L [49]), although not specifically considering IDH as an outcome.

### Ultrafiltration rates

To our best knowledge, no RCTs have been conducted to show that avoiding higher ultrafiltration rates can systematically decrease IDH. Ultrafiltration rates are a function of ultrafiltration goals (dictated by target weight) and treatment time, slower ultrafiltration being associated with better survival [50], and faster ultrafiltration (higher ultrafiltration rates through higher ultrafiltration goals and/or shorter treatments) being associated with higher mortality [51, 52]. Suggesting that dialysis physicians should avoid higher ultrafiltration rates to avoid IDH makes intuitive sense but has not been formally proven.

### Dialysate temperature

In an observational study of 8071 incident dialysis patients, a 0.5°C decrement in the dialysate temperature was associated with a 2.4% lower risk of IDH (OR 0.976, 95% CI 0.957–0.995) [53], and cooler dialysate temperature was associated with lower risk of IDH (OR 0.67, 95% CI 0.63–0.72) in an adjusted case-mix analysis [53]. The MyTEMP trial [54]—a large, cluster-randomized clinical trial across 84 dialysis centers over 4 years—found that personalized cooler dialysate temperature did not improve the primary endpoint of cardiovascular events and did not lead to a significant drop in intradialytic systolic BP, but IDH frequency was modestly lower: the proportion of treatments with IDH (Nadir90 criteria or a drop in systolic BP >25% from pre-dialysis) was 30.4% among the cooler dialysate group compared with the 32.4% in the standard temperature group (mean difference −2.0%, 99% CI −3.6, −0.4). In an updated 2025 meta-analysis of small, randomized trials, the use of a low dialysate temperature was associated with a significant reduction in the incidence of IDH (OR 0.35, 95% CI 0.28–0.46) [55]. Thus, several sources support the notion that lowering the dialysate temperature is an effective strategy to prevent the frequency and severity of IDH.

### Evidence level

The evidence for this tip is heterogeneous. Dialysate sodium: meta-analysis of randomized trials, with a countervailing effect on interdialytic weight gain and a large cluster-randomized trial of hard outcomes still awaited. Ultrafiltration rate: observational data and physiological reasoning only. Dialysate temperature: a large cluster-randomized trial together with meta-analyses of small, randomized trials.

## TIP 6 (PREVENT): INDIVIDUALIZE THE DIALYSIS SCHEDULE

Shorter dialysis treatments were associated with higher long-term cardiovascular and mortality risk [56], as they require more aggressive ultrafiltration rates, risking hemodynamic instability [57]. Shorter delivered dialysis does not only trigger IDH but is also associated with volume excess and more difficult-to-control hypertension, particularly in patients with a high dialysis vintage without residual diuresis. A post-hoc analysis of the DRIP trial showed that shorter duration of the delivered dialysis was associated with higher levels of interdialytic ambulatory systolic BP [58], and that dry weight probing combined with 4-h sessions improved interdialytic ambulatory systolic BP compared with shorter dialysis sessions [58].

In IDH-prone patients, extending the dialysis duration is a mechanistically plausible approach to avoid aggressive ultrafiltration rates and subsequently minimize severity and frequency of IDH [59]. In a crossover trial enrolling 38 dialysis patients, extending dialysis sessions to 5 h was associated with a significantly lower incidence of IDH over a 2-week period as compared with standard 4-h dialysis treatments [60]. IDH-prone patients may also benefit from switching to more intensive dialysis regimens, such as short-daily or thrice-weekly nocturnal dialysis. In a post-hoc analysis of the Frequent Hemodialysis Network (FHN) trial [61], relative to conventional dialysis, short-daily dialysis provoked a greater reduction in pre-dialysis systolic BP (between-group difference: −7.7 mmHg, 95% CI −11.9 to −3.5 mmHg) over the first 2 months of follow-up. A similar improvement in pre-dialysis systolic BP was observed over 2 months of extended-duration nocturnal dialysis (between-group difference: −7.3 mmHg, 95% CI −14.2 to −0.3 mmHg) [61]. Notably, this clinically meaningful improvement in BP control was achieved with a parallel reduction in the number of prescribed antihypertensive medications as well as with an additive reduction in the incidence rates of IDH [59, 61].

An important first step is to avoid shorter dialysis treatments and prescribe adequate dialysis in IDH-prone patients. When patients remain unresponsive to the standard-of-care dialysis schedule, an individualized approach that includes long treatment times or more frequent dialysis sessions should be considered.

### Evidence level

This tip is based on one small crossover trial of session length, post-hoc analyses of a randomized trial of dialysis frequency and observational data on treatment time, and is thus mechanistically coherent but not established for IDH as a primary endpoint.

## TIP 7 (DEFINE AND MONITOR): ROUTINELY MEASURE BLOOD PRESSURE INTRADIALYSIS AND CONSIDER AMBULATORY BLOOD PRESSURE

Intradialytic BP measurement frequency varies by region and provider, typically ranging from two per hour [2, 7] to pre- and post-dialysis only, with intradialytic readings only following symptoms. Convincing evidence on a preferred intradialytic measurement frequency, however, is lacking [62]. In a retrospective analysis of 4348 patients from 21 US dialysis units, Keane *et al.* [2] found that IDH episodes (BP measured every 30 min) were homogenously distributed across the dialysis treatment using the Nadir90 criteria. Crucially, the Nadir90 criteria do not discriminate by the number of IDH episodes per treatment, but only whether or not the criteria were met at any timepoint during the treatment [7]. A recent study of 40 hemodialysis patients compared quarter-hourly brachial BP readings with continuous BP measurements using a photoplethysmographic finger cuff [63]. Brachial cuff readings met the Nadir90/Nadir100 criteria for IDH in 4.5% of measurements compared with 8.0% of finger cuff measurements, but the systematic underestimation of BP by the finger cuff device was noted as a critical limitation.

Evidence for interdialytic BP monitoring in IDH-prone patients is sparse. A single observational study of 36 hemodialysis patients using 48-hour ambulatory BP measurements was conducted on this topic, which found that 5.6% of BP readings within the first 4 h post-dialysis indicated hypotension (notably defined by a drop of systolic BP below 110 mmHg, or a decline below 80% of the mean) compared with 1.6% during any other period [64]. That said, home/ambulatory BP can help distinguish two important subgroups: patients with IDH and uncontrolled interdialytic hypertension, versus patients with IDH and adequately controlled home BP. In the latter group, there is stronger justification for increasing target weight and reducing antihypertensive therapy to prevent IDH. In those with uncontrolled hypertension outside of dialysis, other interventions are perhaps more suitable for the prevention of IDH.

Thus, we recommend routinely measuring BP as often as possible (and acceptable by patients; typically 2/hour in unstable patients and 1/hour in stable patients) throughout the intradialytic period by means of brachial cuff measurements. Ambulatory BP measurements are recommended for all hemodialysis patients to unmask spurious interdialytic or diurnal patterns, though no evidence specifically supports 44-hour measurements for IDH.

### Evidence level

This tip is based on observational data and expert opinion. No trial has compared measurement schedules. The frequencies we suggest reflect the sampling used in the cohorts from which the Nadir90/Nadir100 criteria were derived.

## TIP 8 (PREVENT): CONSIDER HEMODIAFILTRATION?

A consensus statement from the EuDial Working Group of the ERA concluded that, based on available RCT evidence [65–68], online hemodiafiltration (HDF) does not reduce IDH frequency compared with high-flux hemodialysis [69]. Results versus low-flux hemodialysis remain inconsistent [70].

In the ESHOL (On-Line Hemodiafiltration Survival) study, HDF was associated with less frequent symptomatic IDH than high-flux hemodialysis (HR 0.72, 95% CI 0.68–0.77), though symptomatic hypotension was undefined [65]. The FRENCHIE study in 381 elderly patients found no significant reduction in symptomatic hypotension with HDF versus high-flux hemodialysis, though asymptomatic IDH (fall in systolic BP >20 mmHg) was less frequent with HDF [66]. The Turkish Study found no significant difference between treatment groups (*P* = .9) using a definition of IDH based on systolic BP decrease >30 mmHg combined with the need for saline infusion [67]. Locatelli *et al.* compared standard hemodialysis, online predilution hemofiltration and online predilution HDF in 146 patients: symptomatic hypotension decreased significantly with hemofiltration (OR 0.69) and HDF (OR 0.46) [71]. In a four-arm crossover study, Rootjes *et al.* found HDF with high convective volumes (>23 L/1.73 m^2^/session) associated with the lowest hypotensive episode frequency (Nadir90 criteria); lower dialysate temperature also improved hemodynamic stability, though with more temperature-related side effects [72]. In contrast, Smith *et al.* reported a higher incidence of IDH during HDF (HR 1.52, 95% CI 1.2–1.9) than during hemodialysis, using a composite definition (>20 mmHg fall in systolic BP plus clinical intervention) [73]. In a crossover RCT, Buchanan *et al.* found no significant difference between HDF and high-flux hemodialysis with cooled dialysate in terms of intradialytic cardiac output, contractility or myocardial perfusion assessed by cardiac magnetic resonance imaging [74]. Li *et al.* also observed no significant between-modality difference in the change in left ventricular systolic function (61.7 ± 11.1% to 62.7 ± 11.2%; *P* = .19), despite a significant within-treatment reduction during predilution HDF (61.3 ± 9.9% to 59.0 ± 10.4%; *P* = .016) [75].

In conclusion, current evidence does not support online HDF as a strategy for preventing IDH. Although some studies have reported fewer hypotensive episodes with HDF (given sufficient convective volumes, and typically in post-dilution mode), findings across trials are inconsistent, hence the choice for online HDF should be guided by the overall positive effects on survival and quality of life rather than by an expectation of a protective effect against IDH [69].

### Evidence level

This tip is based on several randomized trials with inconsistent results and a working-group consensus statement. This is the tip with the most randomized evidence, and that evidence is largely negative for IDH.

## TIP 9 (PREVENT): AVOID FOOD INTAKE DURING DIALYSIS IN HYPOTENSION-PRONE PATIENTS AND PROMOTE EXERCISE

Decreased splanchnic capacity is an important cardiovascular compensatory mechanism in hypovolemia, maintaining effective circulating blood volume [76, 77]. Food intake during hemodialysis counteracts this by causing a blood shift to the splanchnic vasculature via vasorelaxation [78]. Several interventional studies have shown that BP decreased more during treatments with intradialytic food intake than without [78–81]. An observational study showed that intradialytic food consumption of >200 kcal was associated with a 2-fold higher incidence of hypotension [82]. Alternatives include eating after dialysis cessation and increasing caloric and protein intake during the out-of-dialysis period [81].

Intradialytic exercise safely improved physical function and quality of life, and dialysis-related symptoms commonly associated with IDH (e.g. fatigue and muscle cramping) [83–85]. Relatively small clinical studies suggest that intradialytic aerobic exercise attenuates the fall in BP during hemodialysis [86–89]. Crossover studies showed that intradialytic exercise significantly reduced hemodialysis-induced cardiac stunning [90, 91]. A recent crossover study using intradialytic speckle-tracking echocardiography in 60 patients found that myocardial contractility was better preserved during 30 min of intradialytic aerobic exercise compared with hemodialysis without exercise [92].

There has been concern that intradialytic exercise induces a reduction in relative blood volume. Indeed, Banerjee *et al.* observed hemoconcentration during intra-dialytic exercise, probably as a consequence of fluid shifts from the microvasculature to the interstitial space [93], but the overall effect on BP was positive in most patients [86, 93]. This paradox may be explained by the divergent effects that exercise has on the circulation. Capacitive areas of the circulation are contracted (increasing mean systemic filling pressure) and cardiac output increases, whereas on the other hand relative blood volume falls and peripheral vascular resistance decreases [93, 94]. Interestingly, a crossover study comparing the impact of the timing of intradialytic cycling on BP found that there was no difference in symptomatic hypotension between intradialytic cycling in the first half versus the second half of the dialysis session despite the fact that most patients are more hypovolemic during the second half [95].

### Evidence level

The evidence behind this tip is heterogeneous. For food intake, several small interventional studies with consistent findings plus supporting physiology. For exercise, small randomized and crossover studies, including mechanistic imaging studies; consistent in direction but small in scale.

## TIP 10 (PREVENT): USE FEEDBACK ALGORITHMS AFTER CRITICAL REVIEW OF TREATMENT SETTINGS

Automated feedback control in hemodialysis algorithmically adjusts up to three machine outputs (ultrafiltration rate, dialysate conductivity and dialysate temperature) based on continuously or intermittently measured patient states of ultrafiltration-induced hemoconcentration (relative blood volume), blood line temperatures, dialysate conductivity or arterial BPs [96, 97].

Automatic feedback control is offered by major machine manufacturers (Fresenius, Gambro/Hospal/Baxter, Nikkiso, Braun) using proprietary algorithms based on single- or double-input information (hemoconcentration and temperature or BP) and single (ultrafiltration or temperature) or multiple (ultrafiltration and conductivity or temperature) output control. Intradialytic hypotensive events have been reduced with ultrafiltration control alone [98–101], in combination with temperature control [102], but mostly in combination with so-called conductivity control (but without conductivity sensing) [103–114] using measured hemoconcentration as single input, but also when adding arterial BP as a second input [115, 116]. Other more recent studies failed to show a clear reduction in IDH using ultrafiltration control alone or combined with temperature and conductivity control [117–121]. A meta-analysis of seven RCTs showed that relative blood volume–based feedback control reduced the risk of IDH in hypotension-prone patients [122]. Unlike (variable) ultrafiltration, (high) dialysate conductivity and (cool) dialysate temperature are believed to exert a hemodynamic rather than a direct volume effect stimulating vasopressin secretion and preventing heat-induced volume sequestration in the cutaneous circulation [114, 122, 123]. Lowering dialysate temperature with or without feedback control may therefore reduce the rate of IDH and increase intradialytic mean arterial pressure [124–127]. More recent feedback control concepts to deliver dialysis under isonatric or isotonic treatment conditions in support of physiologic osmoregulation remain to be examined in clinical trials [128, 129].

The overall outcome of automatic feedback control trials for reducing IDH is mixed, but the majority demonstrated effectiveness [127, 129]. Long-term studies are missing, but none of the studies reported adverse events. In a pragmatic approach one could therefore start with relatively simple isothermic temperature control followed by more sophisticated ultrafiltration and conductivity control. Thus, even if the average benefit is small, the technology is safe and worth testing in individual IDH-prone patients.

### Evidence level

The evidence base for this tip is a meta-analysis of seven randomized trials showing benefit in hypotension-prone patients, against several more recent neutral trials.

## CONCLUSIONS

IDH remains a frequent complication of hemodialysis, contributing to cardiovascular morbidity, mortality and symptom burden. We have proposed 10 practical tips of decreasing urgency, organized around three categories: (i) defining and monitoring: adopt a uniform IDH definition such as the Nadir90 criteria and document symptoms; regularly measure intradialytic as well as ambulatory BP; (ii) treating: develop a standard operating procedure for acute events; increase dialysate sodium, and decrease ultrafiltration rates and dialysate temperature; (iii) preventing: regularly assess fluid status; review antihypertensive drugs; consider more frequent, longer or nocturnal dialysis; potentially consider hemodiafiltration; avoid food and consider exercise; try automatic feedback algorithms. No single measure consistently prevents IDH, and each tip should be viewed as one lever among many. In practice, IDH is best managed by a layered, individualized approach that combines rigorous documentation, structured follow-up and a willingness to revisit the prescription each time hemodynamic instability recurs. The patient’s own perspective should anchor this process.

## Data Availability

No new data were generated or analyzed in support of this research.
